# Sequential Extraction of Naringin and Low-Ester Pectin from Naturally Dropped Fruit of Pomelo

**DOI:** 10.3390/ijms26178341

**Published:** 2025-08-28

**Authors:** Bo Yu, Liwen Huang, Yuhan Ding, Ning Zhang, Kexin Li, Yuanbao Jin, Zhihua Wu

**Affiliations:** 1 Sino-German Joint Research Institute, Nanchang University, Nanchang 330047, China; yubo@ncu.edu.cn; 2 College of Food Science and Technology, Nanchang University, Nanchang 330047, China; xbb123@email.ncu.eud.cn (L.H.); 417900230105@email.ncu.eud.cn (N.Z.); likexixixin@163.com (K.L.); 3 School of Stomatology, Nanchang University, Nanchang 330047, China; 413009240025@email.ncu.eud.cn; 4 College of Food Science and Engineering, South China University of Technology, Guangzhou 510641, China; 2009jxncmeet@163.com; 5 State Key Laboratory of Food Science and Resources, Nanchang University, 235 Nanjing Rd. East, Nanchang 330047, China

**Keywords:** naringin, pectin, pomelo, dropped fruit, sequential extraction

## Abstract

Naringin and pectin were innovatively and sequentially extracted from naturally dropped fruit of pomelo (NDFP), optimizing yields via response surface methodology (RSM). Under optimized conditions, naringin was extracted first with 70% ethanol (70 °C, 110 min, SSR 15:1 *v*/*w*), followed by pectin extraction from the residue using pH 1.50 acetic acid (80 °C, 160 min, ratio 40:1 *v*/*w*) naringin yield reached 42.77% with 97.6% ± 0.31% HPLC purity, while pectin yield was 14.32%. Critically, the recovered pectin was identified as low-ester and exhibited significantly superior antioxidant activity compared to commercial pectin. This work establishes an efficient sequential extraction process valorizing waste pomelo drop, yielding high-purity naringin and antioxidant-rich, low-ester pectin.

## 1. Introduction

Pomelo (Citrus maxima), a widely cultivated citrus fruit in Asia, is known for its large size, thick rind, and rich content of bioactive compounds such as flavonoids and pectin [1,2,3,4,5]. China is the world’s largest producer of pomelo, with an annual output consistently remaining at around 5 million tons [6].

During the growth period, pomelo trees naturally undergo a self-regulatory physiological process that results in the shedding of immature fruits, a phenomenon termed physiological dropping of fruit [7]. The natural fruit dropping is a common mechanism in citrus crops, where the tree optimizes its nutrient allocation by forming abscission zones, leading to the detachment of excess fruit before maturity [8]. It has been estimated that 20–30% of pomelo fruits are lost annually due to physiological fruit drop, depending on environmental and agronomic conditions [9]. This represents a significant waste of biomass that currently lacks effective commercial utilization.

Despite their immature development, the naturally dropped fruit of pomelo (NDFP) are rich in a variety of high-value bioactive substances such as flavonoids, essential oils, and pectin.

Among the flavonoids of pomelo, naringin is a bitter flavanone glycoside with recognized antioxidant, anti-inflammatory, and cardiovascular-protective properties [10,11]. And pectin is a complex polysaccharide widely used in the food and pharmaceutical industries for its gelling and stabilizing properties [12,13]. Industrial extraction of these compounds has traditionally focused on mature pomelo peels or other citrus byproducts [14,15].

Techniques such as ultrasound-assisted extraction, enzymatic hydrolysis, and microwave-assisted extraction have been successfully applied to citrus sources [16,17]. For example, Harsh P. Sharma et al. found that the use of a complex enzyme method involving cellulase, pectinase, amylase, etc., can provide a higher yield of juice and juice of higher quality [18]. Filipa A. Fernandes et al. used response surface optimization for ultrasonic-assisted extraction of citric acid from citrus peels [19].

Response Surface Methodology (RSM), a powerful statistical tool for optimizing complex processes, has been successfully applied to enhance the extraction of pectin from various citrus and other fruit wastes [20,21]. For example, Olugbenga Abiola Fakayode et al. used the response surface method to study the influence of process parameters on the extraction of orange peel essential oil and pectin. Eventually, the yield of essential oil was 3.38%, and the yield of pectin was 30% [22]. V. Vathsala et al. studied the use of ultrasound-assisted extraction optimization to extract pectin from pomelo and sweet lemon peels and obtained the optimal optimization conditions through RSM [23].

However, current industrial practices predominantly focus on extracting these compounds from mature pomelo peel, while NDFP remains an underutilized source, often discarded or used as low-value animal feed or compost. The application of RSM to optimize the extraction of naringin and pectin specifically from NDFP has not been reported in the literature to date.

The present study addresses this gap by developing a sustainable sequential extraction strategy to obtain high-purity naringin and antioxidant-rich low-ester pectin from NDFP. RSM was applied to systematically optimize critical factors in each extraction stage, targeting maximum yield and optimal functional quality. Compared with prior studies focusing on single products from mature pomelo peel, our approach (1) valorizes the untapped byproduct of pomelo, NDFP. (2) integrates two high-value products in a single processing chain, and (3) enhances process efficiency for potential industrial application. This work contributes to circular economy principles in citrus processing by transforming agricultural waste into valuable bioactive compounds with broad food and pharmaceutical potential.

## 2. Results and Discussion

### 2.1. Effects of Extraction Conditions on Extraction Efficiency

**Figure 1:**
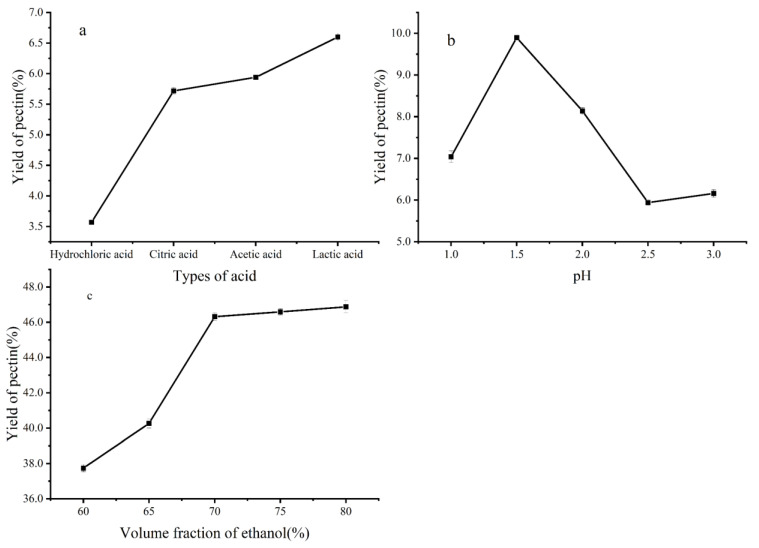
Effects of different factors on the extraction yield: (**a**) Type of acid (pH = 2.5) on the extraction yield of pectin. In order to make the pH of each acid solution is 2.5 at room temperature, the concentrations of various acids are as follows: Hydrochloric acid 3.2 mmol/L, Citric acid 16.5 mmol/L, Acetic acid: 560.0 mmol/L, Lactic acid: 75.0 mmol/L, (**b**) pH on the extraction yield of pectin, (**c**) Volume fraction of ethanol on the extraction yield of naringin.

As shown in Figure 1a, organic acids were the most effective for pectin extraction. Among these six acid types, lactic acid was the most effective for pectin extraction from NDFP. However, acetic acid is a safe edible acid and is less expensive than lactic acid. Therefore, acetic acid was selected for further use. pH is one of the most crucial parameters affecting the amount and properties of extracted pectin, whose yields are high at low pH values. The maximum pectin yield was obtained at pH 1.5 (Figure 1b). When the ethanol concentration reached 70%, the extraction yield of naringin was the highest, up to 46.32%. However, when the ethanol concentration was too high, the extraction ability of ethanol for other alcohol-soluble impurities in the solution system also increased, leading to the intensified competition between other impurities and naringin for the solvent. As a consequence (Figure 1c), the extraction yield of naringin decreased. Therefore, a 70% volume fraction of ethanol was selected as the extractant in this experiment.

### 2.2. Response Surface Optimization of Naringin Extraction Conditions from NDFPs

#### 2.2.1. Statistical Analysis and Model Fitting of Naringin

RSM was used to evaluate the effects of temperature (X_1_), extraction time (X_2_), and SSR (X_3_) on the extraction yield of naringin from NDFPs. According to the test scheme and results in Table S3, the quadratic model was obtained as follows:

$$\begin{array}{l} \mathrm{Naringin}\;\mathrm{Yield}(\%)=+39.67-0.59X_{1}+0.97X_{2}+0.43X_{3}+0.22X_{1}^{2}+0.16X_{2}^{2}+0.23X_{2}^{3}\\ +0.59X_{1}X_{2}+1.45X_{1}X_{3}-0.44X_{2}X_{3} \end{array}$$

where X_i_ is a coded independent factor (X_1_ = temperature, X_2_ = time, and X_3_ = SSR).

As shown in Table S3, analysis of variance revealed that the second-order regression was statistically significant (*p* < 0.05), and the quadratic polynomial model was in good agreement with the test results. In addition, the *p*-values were all lower than 0.05. These results showed that these items had significant effects on the extraction yield of naringin from NDFPs. Among these items, X_1_, X_2_, and X_1_X_3_ were extremely significant, indicating that temperature, time, and the interaction between temperature and SSR had the most significant effect on the extraction yield of naringin. According to the F value, the effects of these three factors on the extraction yield of naringin were in the following order: X_2_ (extraction time) > X_1_ (temperature) > X_3_ (SSR). The misfit item was not significant, indicating that the model is accurate enough to predict naringin yield for any combination of independent factors within the scope of this study. The coefficient of determination (R^2^) of naringin extraction yield was as high as 0.9892. The adjusted R^2^ was 0.9753, indicating that the model is highly significant. Therefore, the obtained model can be used to determine the relative effects of factors to achieve the best combination of parameters for high naringin yield and to predict test results under other conditions.

#### 2.2.2. Analysis of Response Surfaces

**Figure 2:**
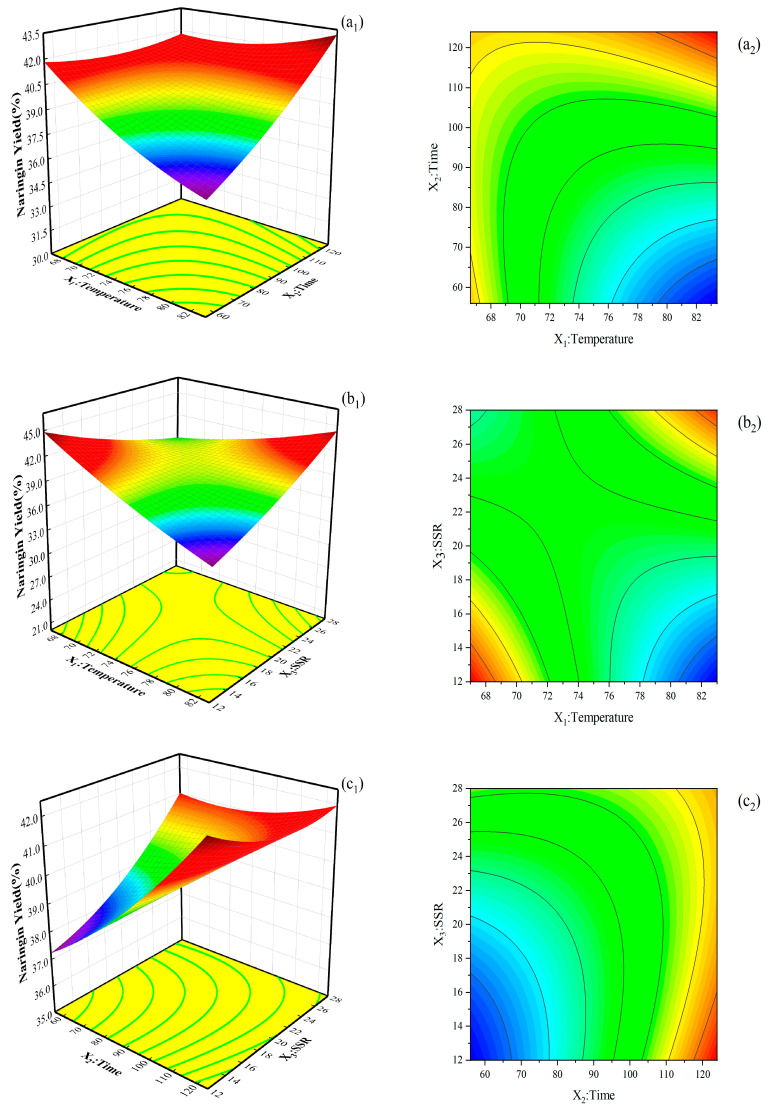
3-D response surface plots (**left**) show the effects of process parameters on the naringin extraction yield for the interactions between (**a_1_**) temperature and time, (**b_1_**) temperature and SSR, and (**c_1_**) time and SSR; RSM contour plots (**right**) show the interaction effects between (**a_2_**) temperature and time, (**b_2_**) temperature and SSR, and (**c_2_**) time and SSR on the naringin extraction yield.

The interaction of independent variables on the extraction yield of naringin is shown in Figure 2, and an interaction was observed among the factors. The extraction yield of naringin was negatively correlated with temperature, extraction time, and SSR. In particular, the extraction yield of naringin decreased with the increase in temperature. Although the solubility and diffusion coefficient of soluble components increase with the temperature, which is beneficial to naringin extraction, too high a temperature damages the effective components that are not sensitive to heat. In addition, the extractant was seriously volatilized. As a consequence, the extraction yield of naringin was reduced. With the extension of time, the increasing trend of naringin extraction yield slowed down. When the extraction time was 110 min, the extraction yield reached its peak. If the extraction time is further extended, the extraction yield will not continue to increase but will instead decrease. This is because the phenolic hydroxyl groups in naringin were prone to undergo oxidation reactions when exposed to high temperatures for a long period of time. Additionally, an excessively long extraction time will also cause some of the ethanol in the extraction agents to evaporate. With the increase in the SSR, the contact area between fruit drop and extractant and mass transfer efficiency also increased. However, when the SSR exceeded 15:1 *v*/*w*, naringin fully dissolved, and its content no longer increased with the SSR. Ethanol extraction involves mass transfer and diffusion, which take a certain amount of time. Increasing the amount of solvent leads to failure to complete the mass transfer in a certain extraction time. As a consequence, the extraction yield of naringin decreases slightly.

With the use of the regression equation model and by taking the maximum response value as the optimization index of the extraction, the optimum theoretical conditions for extracting naringin from NDFPs were obtained as follows: temperature is 70 °C, extraction time is 110 min, and SSR is 15:1 *v*/*w*. Under these conditions, the extraction yield of naringin was 42.71%. Three parallel experiments were carried out under these conditions to verify the effectiveness of the model, and the average total extraction yield was 42.77%, which was not much different from the predicted value. Therefore, the mathematical model for optimizing naringin extraction from NDFPs by CCD RSM is accurate and reliable.

### 2.3. Response Surface Optimization of the Extraction Conditions of Pectin from NDFPs

#### 2.3.1. Statistical Analysis and Model Fitting of Pectin

RSM was used to evaluate the effects of temperature (X_1_), extraction time (X_2_), and SSR (X_3_) on the yield of pectin from NDFPs. According to the test scheme and results in Table S4, the quadratic model was obtained as follows:

$$\begin{array}{l} \mathrm{Pectin}\;\mathrm{Yield}(\%)=+6.53+1.52X_{1}+0.31X_{2}+0.27X_{3}+0.46X_{1}^{2}+0.13X_{2}^{2}+0.012X_{3}^{2}\\ +0.11X_{1}X_{2}-0.058X_{1}X_{3}-0.058X_{2}X_{3} \end{array}$$

As shown in Table S4, analysis of variance revealed that the second-order regression was statistically significant (*p* < 0.05), and the quadratic polynomial model was in good agreement with the test results. In addition, the *p*-values of the linear coefficients (X_1_, X_2_, and X_3_) and quadratic coefficients were all lower than 0.05, indicating that these items had a significant effect on the extraction yield of pomelo pectin. Among these items, X_1_ was extremely significant, indicating that temperature has the most significant effect on the yield of pectin. According to the F values, the effects of these three factors on pectin yield were in the following order: X_1_ (temperature) > X_2_ (extraction time) > X_3_ (SSR). The misfit item was not significant, indicating that the model is accurate enough to predict pectin yield for any combination of independent factors within the scope of this study. The coefficient of determination (R^2^) of the yield was as high as 0.9923. The adjusted R^2^ was 0.9825, indicating that the model is highly significant. Therefore, the obtained model can be used to determine the relative effects of factors to achieve the best combination of parameters for high pectin yield and to predict test results under other conditions.

#### 2.3.2. Analysis of Response Surfaces

**Figure 3:**
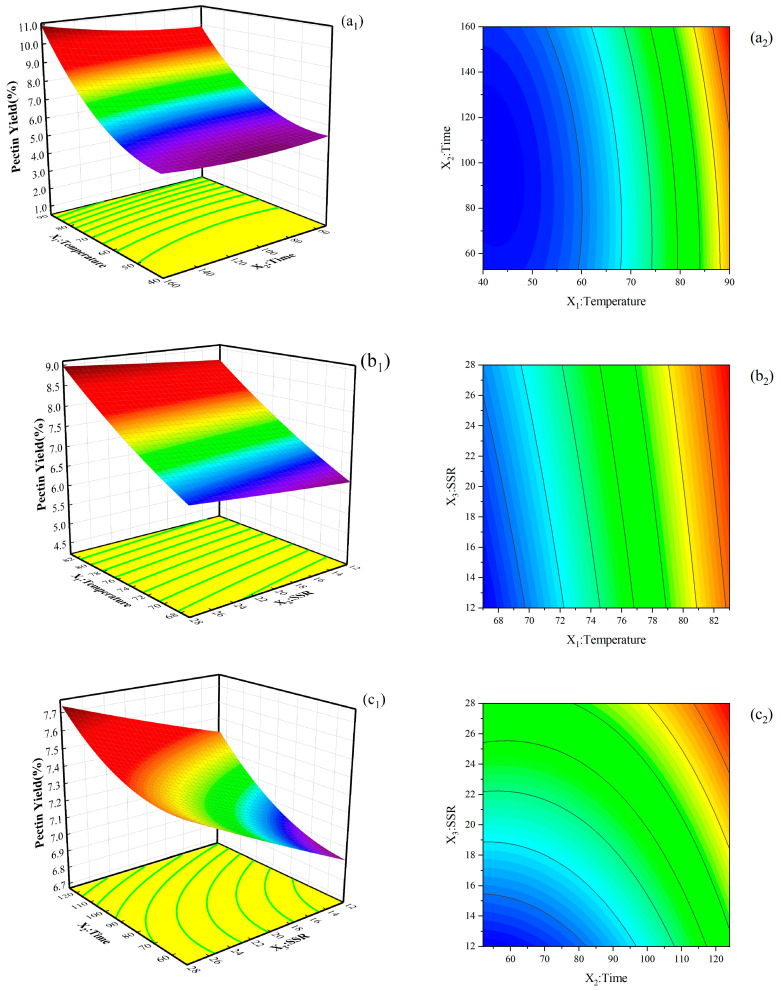
3-D response surface plots (**left**) show the effects of process parameters on the pectin extraction yield for the interactions between (**a_1_**) temperature and time, (**b_1_**) temperature and SSR, and (**c_1_**) time and SSR; RSM contour plots (**right**) show the interaction effects between (**a_2_**) temperature and time, (**b_2_**) temperature and SSR, and (**c_2_**) time and SSR on the pectin extraction yield.

Table S1 shows that the pectin yield varied between 4.84% and 10.30%. Figure 3 illustrates the interactive effect of independent variables on pectin yield, two of which were fixed. The yield of pectin increased with temperature, time, and SSR. This phenomenon may be due to the increase in the solubility and diffusivity of pectin from plant cells to solvents with the increase in temperature. The longer the contact time, the greater the mass transfer of solid particles into the solution. The high SSR increases the yield by increasing the contact surface between plant cells and solvents and promotes the expansion of plant cells, resulting in cell rupture. As a consequence, the dissolution of pectin is enhanced. These results are consistent with those obtained in sour orange peel by Colodel et al. [24].

By using the regression equation model and taking the maximum response value as the index of optimization of the extraction, the optimum conditions of extracting pectin from NDFPs with acetic acid were obtained as follows: temperature is 80 °C, the time is 159.97 min (the real experimental time is adjusted to 160 min), and the SSR is 40:1 *v*/*w*. Under these conditions, the extraction yield of pectin was 9.23%. Three groups of parallel experiments carried out under these conditions revealed that the average total extraction yield was 9.31%, which was not much different from the predicted value.

Through this extraction route, the adverse effect of naringin on pectin extraction was avoided, leading to an effective improvement in the yield and quality of pectin.

Under the optimal processing conditions, the amounts of naringin and pectin obtained from the NDFP (dry weight) were 42.77 mg/g and 143.2 mg/g, respectively.

### 2.4. Purity of Naringin

**Figure 4:**
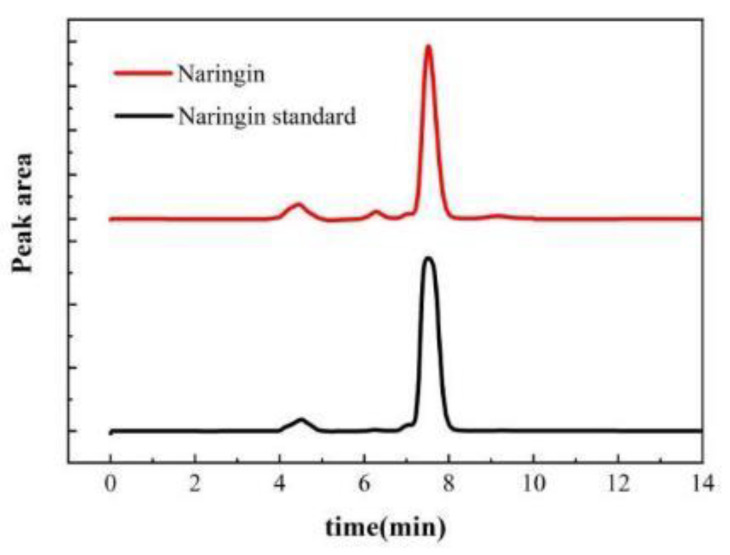
HPLC chromatogram of naringin.

Comparison of the peak time of the liquid phase diagram revealed that the main flavonoid in NDFPs was naringin with a purity of 86.3%. After purification, the purity of naringin reached 97.6% by HPLC analysis (Figure 4). This extraction purity is higher than that reported by Liu [25], reaching more than 95%. These results show that this traditional purification method can purify naringin in large quantities and with high purity.

### 2.5. Characterization of NDFP Pectin

#### 2.5.1. Basic Properties of Pectin

The acid-insoluble ash content of pectin from NDFPs reached the national standard. Its DE was 32.57% ± 0.09%, classifying it as a low-ester pectin. This DE is lower than that of commercial pectin by 60–70%. Although extraction might change DE, no extraction in our work could hydrolyze sugar from pectin. Therefore, the pectin in immature pomelo is believed to naturally have a low DE. The Gal-A content in the pectin sample was 85.86% ± 0.02%, and the protein content was 1.61% ± 0.01%, which is significantly higher than that in standard pectin. According to its intrinsic viscosity, the MW of the pectin sample was approximately 69.42 kDa (Table 1). These results show that the pectin extracted by this method is better than the standard pectin purchased.

#### 2.5.2. Emulsifying Properties

The obtained pectin exhibits good emulsifying properties. Under the optimum extraction conditions, the stability of the emulsion stored at 4 °C and 23 °C for 1 and 30 days, respectively, was evaluated. The emulsifying activity reached 46.97% ± 0.38% on the 23rd day. When stored at 4 °C, the emulsifying stability decreased from 90.30% ± 0.13% on the 1st day to 89.14% ± 0.03% on the 30th day. When stored at a room temperature of 23 °C, the emulsifying stability decreased from 77.42% ± 0.16% on the 1st day to 77.40% ± 0.07% on the 30th day. This finding indicates that the pectin sample exhibits good emulsifying stability within a month. The emulsifying property and emulsifying stability of the pectin extracted under this route are better than those reported by Raji Z. [26]. The pectin sample extracted from the filter residue was characterized as low-ester pectin, and its emulsifying property and stability are higher than those of standard pectin.

### 2.6. Analysis of Scanning Electron Microscope

**Figure 5:**
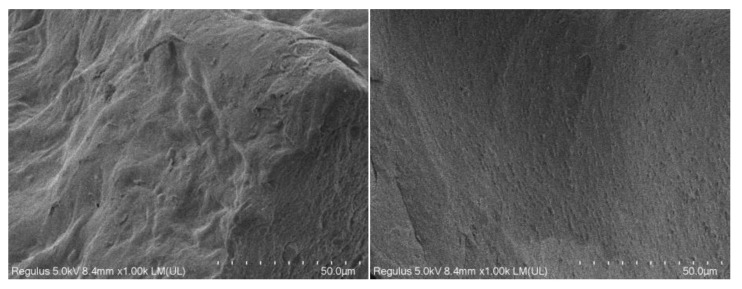
Morphology of pectin samples (**left**) and pectin standard (**right**).

The SEM images of the two pectin samples are displayed in Figure 5. The standard pectin showed puffy and soft folds with sporadic particles stuck on the surface (Figure 5 right). However, the extracted pectin sample (Figure 5 left) exhibited a rather rough structure with a bumpy, uneven surface. The different appearances of the two pectin samples could be attributed to the differences in their structures.

### 2.7. Antioxidant Capacity of Pectin

**Figure 6:**
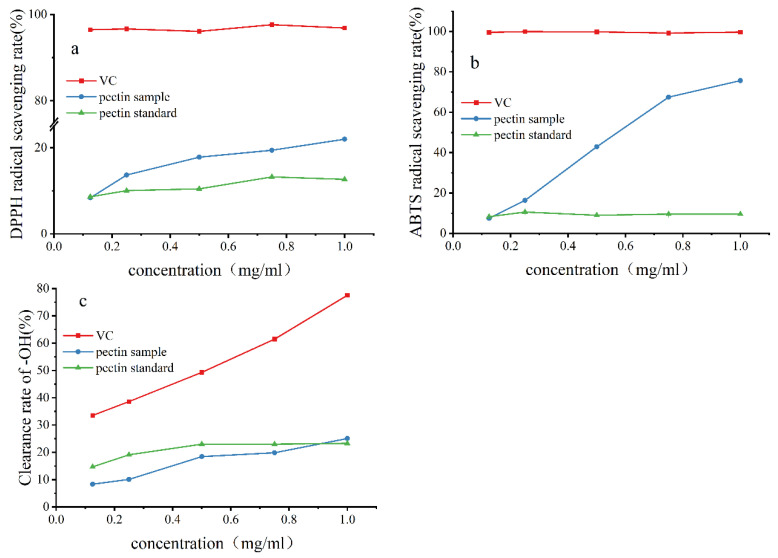
The scavenging ability of pectin from NDFP for various free radicals. (**a**) The free radical scavenging ability for DPPH; (**b**) The free radical scavenging ability for ABTS; (**c**) The free radical scavenging ability for ·OH.

The extracted pectin sample had higher antioxidant activity than the standard pectin. The antioxidant activities of the two kinds of pectin (pectin sample, pectin standard) were compared by DPPH, ·OH, and ABTS scavenging experiments (Figure 6). The free radical scavenging ability of the two increased with their concentration. The antioxidant activity of the pectin sample was stronger than that of the pectin standard, but both had lower antioxidant activity than vitamin C. The effect of low pectin concentration on ·OH scavenging was not good. A possible reason is that the concentration of pectin did not reach the concentration needed to capture ·OH in competition with salicylic acid, which was dominating the competition. When the mass concentration reached 1.0 mg/mL, the ·OH scavenging rate of the pomelo pectin sample (25.07%) was slightly higher than that of the standard pectin (23.28%). With the increase in the concentration of pomelo pectin, its ·OH scavenging effect will be continuously enhanced.

Based on the sequential extraction process developed in this study, naringin (42.77% yield, 97.6% purity) and LMP (14.32% yield) were successfully co-extracted from NDFP, demonstrating an efficient valorization strategy for underutilized agricultural waste. The sequential approach, first extracting naringin with 70% ethanol (70 °C, 110 min, SSR 15:1 *v*/*w*) followed by pectin extraction from the residue using acetic acid (pH 1.50, 80 °C, 160 min, SSR 40:1 *v*/*w*), eliminated interference between target compounds and maximized yields. Critically, the recovered pectin exhibited a low degree of esterification (DE = 32.57%) and superior bioactivity: it outperformed commercial pectin in antioxidant capacity and emulsifying stability (>89% retention after 30 days at 4 °C). This work establishes a sustainable, industrially viable pathway to transform NDFP into high-purity nutraceuticals (naringin) and functional food additives (low-ester pectin), addressing both waste management and economic challenges in the pomelo industry.

Based on the sequential extraction of high-purity naringin and antioxidant-rich LMP from NDFP demonstrated in this study, future research should carry on the advanced green extraction techniques (e.g., enzyme-assisted, subcritical water, or ionic liquids) that warrant optimization to enhance yield efficiency while minimizing chemical usage, particularly for scaling LMP production. Purification scalability must be addressed through novel methods like membrane filtration or chromatographic separation to achieve > 99% naringin purity economically. Application research in low-sugar gels, nutraceutical co-delivery systems, and active packaging could transform these extracts into market-ready products. Finally, integrated biorefinery models should be developed to valorize residual pomelo components (e.g., lignin, cellulose) post-naringin/pectin extraction, ensuring zero-waste circularity.

## 3. Materials and Methods

### 3.1. Materials and Chemicals

The NDFP used in this study were collected from a local fruit orchard of Ganzhou, Jiangxi Province, China, in the middle of May. The NDFPs and their cross-sectional view are shown in Figure S1. The average cross diameter of the NDFPs ranges from 4.0 to 5.5 cm, and their average weight ranges from 52 to 55 g.

Glacial acetic acid, hydrochloric acid, sulfuric acid (superior purity), NaOH, H_2_O_2_, and anhydrous ethanol were purchased from Xilong Science Co., Ltd., Shantou, China. Citric acid, lactic acid, potassium persulfate, ferrous sulfate heptahydrate, salicylic acid, ascorbic acid (V_C_), and naringin were bought from Sangon Biotech (Shanghai) Co., Ltd., Shanghai, China. Phenolphthalein, carbazole, 1-diphenyl-2-trinitrophenylhydrazine (DPPH), and ABTS were acquired from Shanghai McLean Biochemical Technology Co., Ltd., Shanghai, China. PC-300 liquid biological preservative and 5 × Coomassie brilliant blue GMUE 250 were obtained from Beijing Solebo Technology Co., Ltd., Beijing, China. D-galacturonic acid, bovine serum albumin, and pectin were provided by Aladdin Biochemical Technology Co., Ltd., Shanghai, China.

All other chemicals and reagents were of analytical grade and purchased locally.

### 3.2. Extraction of Naringin and Pectin

The NDFP (the whole fruit without separation) was sliced and dried in an oven (DHG, Shanghai Xinmiao Medical Devices Manufacturing Co., Ltd., Shanghai, China) at 60 °C until constant weight and then crushed into powder (particle size: 0.4 mm) with an electric multifunctional pulverizer (400Y, Shandong Mobeli Powder Technology Equipment Co., Ltd., Weifang, China). The obtained powder was stored in a dry environment prior to the experiment. At a certain solvent-to-solid ratio (SSR) and volume fraction of ethanol, the NDFP powder was treated by heating reflux at a certain temperature and extraction time. After being cooled to room temperature, the extract was filtered, and the filter residue was collected for secondary extraction. The alcohol extract of flavonoids was obtained. After flavonoids were extracted, the NDFP filter residue was washed with water so it does not have any alcoholic flavor, dried in an oven at 60 °C to a constant weight, and sifted through a 40 mesh.

The yield of naringin was calculated according to the following formula.


(1) $$\mathrm{Yield}(\%)=\frac{\mathrm{weight}\;\mathrm{of}\;\mathrm{dried}\;\mathrm{extracted}\;\mathrm{naringin}\;\left(g\right)}{\mathrm{weight}\;\mathrm{of}\;\mathrm{dried}\;\mathrm{NDFP}\;\mathrm{power}(g)}\times 100$$

Pectin was obtained by heating the NDFP filter residue and acid solution in a water bath. The hot extracting solution was filtered through two layers of gauze, and the filtrate was centrifuged at 5000× *g* for 30 min. The supernatant was precipitated with twice the volume of ethanol (96%) at 4 °C for 24 h. The precipitate was washed with ethanol twice to remove the alcohol-soluble impurities. Finally, the precipitated pectin was dried under vacuum (DZF-6020, Shanghai Yiheng Scientific Instruments Co., Ltd., Shanghai, China) at 50 °C to a constant weight.

The yield of extraction was calculated according to the following formula.

(2) $$\mathrm{Yield}(\%)=\frac{\mathrm{weight}\;\mathrm{of}\;\mathrm{dried}\;\mathrm{extracted}\;\mathrm{pectin}\;\left(g\right)}{\mathrm{weight}\;\mathrm{of}\;\mathrm{dried}\;\mathrm{NDFP}\;\mathrm{power}(g)}\times 100$$

### 3.3. Optimization of Naringin and Pectin Extraction

The optimum ethanol concentration of naringin and the most suitable acid and pH of pectin were optimized by single-factor experiments. The concentration of ethanol ranged from 60% to 80%. The effect of acid types such as citric acid, acetic acid, lactic acid, and hydrochloric acid was determined. The pH of pectin ranged from 1.0 to 3.0.

On the basis of the single-factor experiment results, the central composite design of response surface methodology (RSM) was used to optimize the extraction parameters of naringin and pectin with three factors and three levels. The experimental design of RSM with factors and their levels is shown in Tables S1 (pectin) and S2 (naringin).

### 3.4. Purification and Identification of Naringin

After the extraction of flavonoids, the alcohol extract was concentrated to 1/10 of the original volume using a rotary evaporator. All the concentrated solutions were stirred in hot water of tenfold volume at 75 °C and filtered quickly while hot. The process was repeated three times to fully remove the impurities. After being allowed to stand at 4 °C for 12 h, naringin was precipitated, and the residue was dried in a vacuum drying oven at 60 °C to obtain refined naringin.

The obtained naringin was characterized by HPLC (LC-15C, Shimadzu, Kyoto, Japan). Chromatographic isolation was carried out on a Shim-pack GWS C18 column (4.6 × 250 mm, 5 μm). The mobile phase was prepared with acetonitrile, water, and acetic acid (30:69.9:0.1, *v*/*v*/*v*). Other conditions for HPLC analysis were as follows: 0.6 mL/min flow rate, 15 μL injection volume, and 30 °C column temperature. Absorbance was monitored at a wavelength of 283 nm with a run time of 15 min. The corresponding calibration curve for naringin was Y = 18,834X + 201,570 (R^2^ = 0.9999). Good linearity was observed for the determination of naringin in the range of 0.1–1.0 mg/mL.

### 3.5. Characterization of Pectin

The content of acid-insoluble ash and D-galacturonic acid of pectin samples was determined in accordance with the National Standard of the People’s Republic of China, “Food Safety National Standard—Food Additives—Pectin” (GB 25533-2010) [27]. The DE of pectin was determined using the titrimetric method described by Pinheiro et al. [28]. The intrinsic viscosity and molecular weight (MW) of the pectin solution were determined with a capillary viscometer [29,30,31].

Following the methods of Li et al. [32] with slight modifications, emulsions were prepared by adding 15 mL of vegetable oil to 15 mL of pectin solution (0.5% *w*/*w*) containing 0.02% PC300 as a bactericide. The mixture was homogenized at 12,000× *g* for 3 min at room temperature in a high-speed dispersion homogenizer (FJ200-SH, Shanghai Huxi Industrial Co., Ltd., Shanghai, China). The samples were centrifuged at 527× *g* for 5 min at 23 °C. After centrifugation, the whole volume of the solution and the emulsified layer volume were determined. Similar emulsion samples were prepared to study emulsion stability (ES) after 1 and 30 days of storage at 4 °C and 23 °C. The samples were centrifuged at 527× *g* for 5 min at these two temperatures. The initial emulsified layer volume was measured. After each storage period, the samples were centrifuged, and the remaining emulsified layer’s volume was measured.

Emulsifying activity (EA) and ES were calculated using the following relations:

(3) $$\mathrm{EA}(\%)=\frac{V_{\mathrm{EL}}}{V_{W}}\times 100$$

V_EL_ is the volume of the emulsification layer; V_W_ is the volume of the whole solution

(4) $$\mathrm{ES}(\%)=\frac{V_{\mathrm{Er}}}{V_{\mathrm{Ei}}}\times 100$$

V_Er_ is the remaining volume of the emulsified layer; V_Ei_ is the initial emulsion layer volume.

### 3.6. Observation Pectin Morphology Using a Scanning Electron Microscope

After extraction, the dried pectin samples and standard pectin powder were used to observe the pectin morphology under a scanning electron microscope (SEM, Quant200, FEI, Tokyo, Japan). Before imaging, the pectin samples were sputtered to cover the gold and palladium layer at room temperature.

### 3.7. Analysis of Antioxidant Activity

The DPPH• scavenging activity of the samples was determined using the reported method of Goulas, V [33]. The total antioxidant activity of two pectins was determined by using an ABTS assay according to the reported method [34]. The scavenging ability of •OH was determined as described previously by Chen [35].

### 3.8. Statistical Analysis

The solvents and chemicals employed in this study were of analytical grade and obtained commercially. All the analyses were carried out in triplicate (*n* = 3), and the data are presented as mean ± standard deviation (SD). Duncan’s test was used for analysis of variance, and the difference was statistically significant. SPSS 20 (Chicago, IL, USA) was applied for data analysis.

## 4. Conclusions

This study successfully established an innovative sequential extraction process for valorizing NDFP, a significant agricultural waste resource. Using Response Surface Methodology, critical parameters for extracting naringin and low-ester pectin were systematically optimized, maximizing yield and functionality. The RSM models demonstrated high accuracy, with coefficients of determination of 0.9892 and 0.9923 for naringin and pectin, respectively, confirming the methodology’s robustness in parameter optimization. The recovered pectin exhibited a low degree of esterification and superior antioxidant activity compared to commercial pectin. Additionally, the pectin displayed excellent emulsifying stability, underscoring its potential for food and pharmaceutical applications. This sequential approach not only maximizes resource utilization by avoiding interference between target compounds but also transforms underutilized NDFP into high-value nutraceuticals and functional ingredients.

## Figures and Tables

**Table 1:** Basic properties of pectin.

Index	Pectin Sample	Pectin Standard
Acid-insoluble ash (%)	3.45 ± 0.03	3.94 ± 0.12
Protein (%)	1.61 ± 0.01	0.35 ± 0.10
Gal-A (%)	85.86 ± 0.02	77.69 ± 0.02
DE (%)	32.57 ± 0.09	70.55 ± 0.22
intrinsic viscosity (Pa·s)	2.11	2.38 ± 0.14
molecular weight (kDa)	69.42	83.24 ± 0.25

## Data Availability

This study includes all collected and analyzed data in the main manuscript and supplementary documents. Any additional inquiries may be directed to the corresponding author.

## Supplementary Materials

The following supporting information can be downloaded at: https://www.mdpi.com/article/10.3390/ijms26178341/s1.
