# Supplementary material for: Sequential Extraction of Naringin and Low-Ester Pectin from Naturally Dropped Fruit of Pomelo
Source: Int J Mol Sci. 2025 Aug 28;26(17):8341. doi: 10.3390/ijms26178341 (PMC12428494; doi:10.3390/ijms26178341)
Supplement: Supplementary file 1 [file ijms-26-08341-s001.zip › Supplementary material.pdf]

Table S1 Pectin extraction response surface test protocol and results

Run	Independent variable						Actual response	Predicted response
	X_1		X_2		X_3		Yield（%w/w）	Yield（%w/w）
	C	A	C	A	C	A		
1	-1	50	-1	80	-1	20	4.84	5.02
2	1	80	-1	80	-1	20	8.14	7.96
3	-1	50	1	160	-1	20	5.52	5.54
4	1	80	1	160	-1	20	8.80	8.91
5	-1	50	-1	80	1	40	5.81	5.8
6	1	80	-1	80	1	40	8.43	8.51
7	-1	50	1	160	1	40	5.81	6.09
8	1	80	1	160	1	40	9.31	9.23
9	-2	40	0	120	0	30	5.50	5.27
10	2	90	0	120	0	30	10.30	10.39
11	0	65	-2	53	0	30	6.38	6.38
12	0	65	2	187	0	30	7.57	7.42
13	0	65	0	120	-2	13	6.14	6.1
14	0	65	0	120	2	47	7.13	7.02
15	0	65	0	120	0	30	6.40	6.53
16	0	65	0	120	0	30	6.58	6.53
17	0	65	0	120	0	30	6.58	6.53

Table S2 Naringin extraction response surface test protocol and results

Run	Independent variable						Actual response	Predicted response
	X_1		X_2		X_3		Yield（%w/w）	Yield（%w/w）
	C	A	C	A	C	A		
1	-1	70	-1	70	-1	15	41.19	41.07
2	1	80	-1	70	-1	15	35.8	35.81
3	-1	70	1	110	-1	15	42.77	42.71
4	1	80	1	110	-1	15	39.95	39.82
5	-1	70	-1	70	1	25	39.82	39.92
6	1	80	-1	70	1	25	40.42	40.45
7	-1	70	1	110	1	25	39.84	39.8
8	1	80	1	110	1	25	42.64	42.7
9	-α	65	0	90	0	20	41.68	41.73
10	α	85	0	90	0	20	39.39	39.37
11	0	75	-α	50	0	20	38.4	38.37
12	0	75	α	130	0	20	42.21	42.26
13	0	75	0	90	-α	10	39.6	39.73
14	0	75	0	90	α	30	41.57	41.46
15	0	75	0	90	0	20	40.11	39.67
16	0	75	0	90	0	20	39.68	39.67
17	0	75	0	90	0	20	39.2	39.67

Table S3 Analysis of variance and coefficient estimate for second-older model (naringin)

Source	Sum of squares	DF	Mean square	F-Value	P-Value
Model	46.23	9	5.14	71.07	<0.0001
X_1	5.55	1	5.55	76.73	<0.0001
X_2	15.13	1	15.13	209.36	<0.0001
X_3	2.99	1	2.99	41.41	0.0004
X_1^2	0.93	1	0.93	12.87	0.0089
X_2^2	0.51	1	0.51	7.00	0.0331
X_3^2	1.04	1	1.04	14.38	0.0068
X_1X_2	2.81	1	2.81	38.36	0.0004
X_1X_3	16.76	1	16.17	231.91	<0.0001
X_2X_3	1.55	1	1.55	21.43	0.0024
Residual Error	0.51	7	0.072		
Lack-of-Fit	0.091	5	0.018	0.088	0.9861
Pure Error	0.41	2	0.21		
Cor Total	46.74	16			
R^2			0.9892		
Adj R^2			0.9753		
Pred R^2			0.9657		

Table S4 Analysis of variance and coefficient estimate for second-older model

(pectin)

Source	Sum of squares	DF	Mean square	F-Value	P-Value
Model	36.64	9	4.07	100.6	<0.0001
X_1	31.6	1	31.6	780.71	<0.0001
X_2	1.3	1	1.3	32.24	0.0008
X_3	1.02	1	1.02	25.1	0.0015
X_1^2	2.38	1	2.38	58.87	0.0001
X_2^2	0.2	1	0.2	4.9	0.0624
X_3^2	0.0018	1	0.0018	0.0434	0.8409
X_1X_2	0.092	1	0.092	2.28	0.1744
X_1X_3	0.026	1	0.026	0.65	0.4454
X_2X_3	0.026	1	0.026	0.65	0.4454
Residual Error	0.28	7	0.04		
Lack-of-Fit	0.26	5	0.052	4.85	0.1799
Pure Error	0.022	2	0.011		
Cor Total	36.92	16			
R^2			0.9923		
Adj R^2			0.9825		
Pred R^2			0.9406		

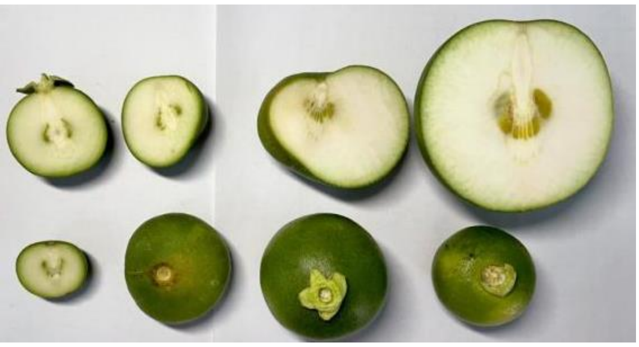

Figure S1. NDFPs and their cross-sections
